# S1P_1_ Receptor Modulation Preserves Vascular Function in Mesenteric and Coronary Arteries after CPB in the Rat Independent of Depletion of Lymphocytes

**DOI:** 10.1371/journal.pone.0097196

**Published:** 2014-05-12

**Authors:** Iryna V. Samarska, Hjalmar R. Bouma, Hendrik Buikema, Hubert E. Mungroop, Martin C. Houwertjes, Anthony R. Absalom, Anne H. Epema, Robert H. Henning

**Affiliations:** 1 Department of Anesthesiology, University of Groningen, University Medical Center Groningen, The Netherlands; 2 Department of Clinical Pharmacy and Pharmacology, University of Groningen, University Medical Center Groningen, The Netherlands; UAE University, Faculty of Medicine & Health Sciences, United Arab Emirates

## Abstract

**Background:**

Cardiopulmonary bypass (CPB) may induce systemic inflammation and vascular dysfunction. Sphingosine 1-phosphate (S1P) modulates various vascular and immune responses. Here we explored whether agonists of the S1P receptors, FTY720 and SEW2871 improve vascular reactivity after CPB in the rat.

**Methods:**

Experiments were done in male Wistar rats (total n = 127). Anesthesia was induced by isoflurane (2.5–3%) and maintained by fentanyl and midazolam during CPB. After catheterization of the left femoral artery, carotid artery and the right atrium, normothermic extracorporeal circulation was instituted for 60 minutes. In the first part of the study animals were euthanized after either 1 hour, 1 day, 2 or 5 days of the recovery period. In second part of the study animals were euthanized after 1 day of postoperative period. We evaluated the contractile response to phenylephrine (mesenteric arteries) or to serotonin (coronary artery) and vasodilatory response to acethylcholine (both arteries).

**Results:**

Contractile responses to phenylephrine were reduced at 1 day recovery after CPB and Sham as compared to healthy control animals (Emax, mN: 7.9±1.9, 6.5±1.5, and 11.3±1.3, respectively). Mainly FTY720, but not SEW2871, caused lymphopenia in both Sham and CPB groups. In coronary and mesenteric arteries, both FTY720 and SEW2871 normalized serotonin and phenylephrine-mediated vascular reactivity after CPB (*p*<0.05) and FTY720 increased relaxation to acetylcholine as compared with untreated rats that underwent CPB.

**Conclusion:**

Pretreatment with FTY720 or SEW2871 preserves vascular function in mesenteric and coronary artery after CPB. Therefore, pharmacological activation of S1P_1_ receptors may provide a promising therapeutic intervention to prevent CPB-related vascular dysfunction in patients.

## Introduction

Cardiopulmonary bypass (CPB) is a widely used technique invaluable to thoracic surgery. CPB is however associated with several side-effects, such as ischemia/reperfusion injury and the induction of a systemic inflammatory response syndrome, potentially leading to multiple organ dysfunction. Several pathological mechanisms are involved in the etiology of CPB-related complications. Contact of blood with the artificial surfaces of extracorporeal circulation circuit (ECC) activates an alternative pathway of complement activation, and induces release of cytokines and inflammatory mediators. Those systemic inflammatory events cause activation of the endothelium, increase its permeability and induce vascular dysfunction with secondary tissue edema and/or tissue hypoperfusion, which sets the stage for multiple organ dysfunction [1]. Although several anti-inflammatory pharmacological agents, including corticosteroids, aprotinin, antioxidants, complement inhibitors and phosphodiesterase inhibitors, have been proposed to inhibit the CPB-related inflammatory processes and vascular dysfunction, none of them substantially improves the clinical outcome of extracorporeal circulation [2].

Vascular reactivity after CPB has been investigated previously in various vascular beds [3]–[7] although follow-up has been limited to only a few hours after CPB. In rat mesenteric artery, 90 min of CPB with 1 and 2.5 h recovery increased the contractile response to phenylephrine and evoked endothelial dysfunction [3], [8]. CPB has been shown to decrease the myogenic reactivity of human skeletal muscles arterioles [7], diminish the contractile response to phenylephrine in human coronary and skeletal arteries [5], [7] and inhibit vasodilatation to acetylcholine in rat cerebral arteries [6]. Abnormality of vascular reactivity in the postoperative period is thought to substantially contribute to the development of organ dysfunction after cardiopulmonary bypass.

FTY-720 (Fingolimod) is a non-selective sphingosine 1-phosphate (S1P) receptor modulator and immunosuppressive agent. Thus far, the therapeutic potential of FTY-720 has been studied in the setting of autoimmune disorders (multiple sclerosis, autoimmune neuritis, autoimmune glomerulonephritis) [9]–[12] and transplantation medicine [12]. Probably, the inhibitory effect of FTY-720 on the migration of lymphocytes, plays an important role in its effect in autoimmune disorders and transplantation medicine [9]–[14]. In addition, FTY-720 exerts diverse pharmacological effects, including vascular effects and modulation of the inflammatory response, via activation of different S1P receptors on endothelial cells, vascular smooth muscle cells, and leukocytes [13].

The vascular effects of FTY-720 are dependent upon the type of vascular bed and the prevalence of the S1P receptor subtypes. It is thought that endothelium expresses S1P1 and S1P3 receptors, which cause NO-mediated vasodilatation. However, S1P2 and S1P3 receptors in vascular smooth muscle mediate a contractile response via the Rho pathway. The systemic hypertensive response evoked by S1P receptors agonists in rodents is thought to be mediated via S1P3 receptors [15]. These, and possibly yet undisclosed effects, result in FTY-720 decreasing ischemia-reperfusion injury [16]–[21], enhancing endothelial barrier function, decreasing vascular permeability [22]–[27] and modulating vascular function various rodent models and endothelial cells [26]–[28]. Therefore, we hypothesize that administration of the S1P receptor agonist FTY-720 will limit vascular dysfunction caused by CPB. Hence, we investigated the effect of FTY-720 on inflammation and vascular dysfunction in a rat model of experimental CPB. First, we characterized the systemic inflammatory response and vascular changes in small mesenteric and coronary arteries induced by CPB during a 5 day follow-up period. Next, we assessed whether FTY-720 and the S1P1R selective agonist, SEW-2871, counteract changes in this CPB model at one day of recovery.

## Methods

### Animals

#### Ethics statement

The protocols for animal care and use were in accordance with the NIH Guide for the Care and Use of Laboratory Animals and approved by the Animal Ethics Committee of the University of Groningen (permit number DEC4714). This study was performed in adult male Wistar rats (body mass 478±52g; Harlan, Zeist, The Netherlands). Animals were housed under standard conditions (i.e. 12∶12 h light:dark cycle and ambient temperature 20–22°C) with free access to food (standard rat chow; Hope Farms, Woerden, The Netherlands) and drinking water throughout the study.

### Experimental groups

#### Vascular effects of CPB

Animals (n = 78) were randomly allocated to either CPB or Sham with a recovery period of either one hour, one day, two or five days. Three animals (4%) were euthanized after uncontrollable bleeding following attempted cannulation. To reveal vascular effects induced by anesthesia and surgery itself, which is expected to occur in both Sham and CPB treated animals, we included naïve untreated rats as additional controls (n = 6). Control animals were sacrificed under isoflurane anesthesia (2.5%). The final number of animals included in the analysis were: CPB 1 hour (n = 13), Sham 1 hour (n = 7), CPB 1 day (n = 11), Sham 1 day (n = 9), CPB 2 days (n = 8), Sham 2 days (n = 8), CPB 5 days (n = 6), Sham 5 days (n = 7) and Control (n = 6).

#### Effect of S1P receptor agonists

Animals (n = 49) were randomly allocated to one of the experimental groups, being either CPB or Sham and to different treatments, being either vehicle, 0.5 mg/kg FTY720 or 0.5 mg/kg SEW2871 injected three hours before the induction of anesthesia. After 1 day of recovery from the procedure animals were euthanized. Thirty seven animals survived and completed the study protocol: CPB FTY720 (n = 6), Sham FTY720 (n = 7), CPB SEW2871 (n = 5), Sham SEW2871 (n = 6), Sham Vehicle (n = 6), CPB Vehicle (n = 7). The remaining 12 animals that did not survive the experimental procedure were equally distributed amongst groups (data not shown). Deaths were mainly due to technical problems (uncontrolled bleeding and cannulation problems).

### Experimental protocol

The experimental protocol consisted of the following parts: anesthesia, preparation, extracorporeal circulation, a weaning and a recovery period that was previously described [29], [30]. Animals that underwent the Sham procedure were subjected to the same procedure as the animals in the CPB group, except for the extracorporeal circulation; these animals maintained mechanically ventilated throughout the procedure.

Anesthesia was induced with 2–3% isoflurane in O_2_/air (1∶1 v/v) before intubation and mechanical ventilation (Amsterdam Infant Ventilator; HoekLoos, Amsterdam, The Netherlands). Tidal volume was set to normocapnia (verified by arterial blood gas analysis), with O_2_/air (1∶2) at a ventilation rate of 50 min^−1^ (0.5 s inspiration time). Rectal temperature was kept at 37.5 ± 0.5 °C, using an electrical heating pad. The left femoral artery was cannulated (26-gauge catheter) for blood pressure monitoring. The mean arterial pressure was kept between 70 and 100 mmHg by adjusting the isoflurane concentration as necessary (typically between 2.0–2.5%). Immediately before insertion of the arterial line, 250 IU kg^−1^ heparin was administered. The left carotid artery was cannulated for arterial inflow using a 22-gauge catheter. A multi-orifice 4.5 French cannula (adapted from a Desilets-Hoffmann catheter, Cook Son, The Netherlands) was advanced into the right heart using the right common jugular vein for access. The tail vein was cannulated for the administration of intravenous anesthetics, heparin, and protamine sulfate.

### Extracorporeal circulation

Subsequently, extracorporeal circulation (ECC) was initiated in the CPB group for 60 minutes. The set-up consisted of a transparent polyvinylchloride venous reservoir, a peristaltic pump (Pericor SF70, Germany), and a neonatal membrane oxygenator (0140GM, Polystan, Vaerlose, Denmark). The oxygenator carried a sterile, disposable three-layer artificial diffusion membrane, made from hollow polypropylene fibers (Jostra AG, Hirrlingen, Germany). All components were connected with polyethylene tubing (1.6 mm inner diameter). The venous reservoir and heat exchanger were sterilized prior to use. The circuit was primed with 15 ml of hydroxyethyl starch 60 mg ml^−1^ solution (Voluven, Fresenius Kabi, Bad Homburg, Germany). Animals were additionally heparinized (250 IU kg^−1^) after the start of ECC. During EEC, rats were anesthetized with intravenous fentanyl (125 µg kg^−1^), atracurium (0.5 mg kg^−1^), and midazolam (2 mg kg^−1^). During CBP, blood oxygen saturation was monitored continuously by a pulse oximeter. Targeted CBP flow was 100–110 mL kg^−1^ min^−1^, corresponding to 60–70% of normal cardiac output.

### Weaning and recovery

During the weaning period, ECC was terminated and mechanical ventilation was initiated. Protamine (150 IU kg^−1^ i.v.) was administered in order to neutralize heparin after which cannulae were removed and the wounds sutured. Following extubation, animals were kept under isoflurane anesthesia (0.8–1.0%) for the first hour of recovery in order to stabilize. Animals were euthanized at the end of the recovery period under isoflurane anesthesia (2.5–3%).

### Blood analysis

Blood gas analysis was performed in both studies and samples (0.1ml) were drawn from the femoral artery at four time-points: at the end of the preparation period (15 min before start of ECC), twice during the ECC period (15 min after the start of ECC and 15 min before its end), and at 15 min after the end of ECC.

In addition, in the second part of the study, blood was taken for full blood counts at the following times and sites: at the time of injection of the compound (FTY-720, SEW-2871, vehicle groups, from the tail vein), at the end of preparation period (15 min before start of ECC, from the tail vein), at 45 min after the end of ECC (from the femoral artery) and at euthanasia (24 hours after ECC, from the aorta).

Blood gas analysis and cell count were performed at the central laboratory of the University Medical Centre Groningen. Mean arterial pressure (MAP) was monitored continuously during the entire protocol via a catheter in a branch of the femoral artery connected to a pressure transducer and a recorder.

### Analysis of plasma IL-6

Plasma interleukin 6 (IL-6), as a marker of the inflammatory response, was determined by Elisa according to the manufacturer's instructions (Rat IL-6 DuoSet, R&D system, Minneapolis, MN, USA). Because of a limitation in the number of samples available and the limited amount of samples that could be handled in one run, a subset of samples was randomly chosen from the following groups: Control (n = 7), Sham-Vehicle 1 hour (n = 6), CPB-Vehicle 1 hour (n = 4), Sham-Vehicle 1 day (n = 5), CPB-Vehicle 1 day (n = 5), Sham 1 hour FTY-720 (n = 4), CPB 1 hour FTY-720 (n = 4); Sham 1 day FTY-720 (n = 2), CPB 1 day FTY-720 (n = 4).

### Vascular reactivity studies

At euthanization, all mesenteric loops and the heart were removed and placed into a cold physiological saline solution. Several segments of the third branch of the mesenteric superior artery and the interseptal coronary artery were dissected, prepared as ring vessel preparations (1.8–2.0 mm in length) and mounted on two 40 µm stainless wires connected to force transducers in individual organ bath chambers for isometric tension recordings in a wire-myograph (Danish Myo Technology A/S, Aarhus, Denmark). The baths contained 6 ml of Krebs solution (pH  =  7.4) warmed to 37°C and perfused continuously with a gas consisting of 95% O_2_ and 5% CO_2_. The vessels were subjected to the standard normalization procedure [31] and left to equilibrate for 40 minutes until they reached a steady baseline. Briefly, arterial segments were subjected to stretching stepwise by increasing the distance between two wires in steps of 10 µm. The target calculated transmural pressure was 100 mmHg for both mesenteric and coronary segments. The values of internal circumference (micrometer reading, µm) and wall tension (force reading, mN/mm) were registered. Then, the normalized internal circumference (set to 0.8 of IC_100_, an internal circumference to a transmural pressure of 100 mm Hg) was calculated for each vascular segment [31]. Thereupon, vessels segments were equilibrated for 40 minutes to reach a steady baseline, followed by two consecutive stimulations with potassium chloride (KCl, 60 mM). Only vessels that demonstrated a reproducible contractile responses to KCl and reproducible contractile responses to the agent employed (phenylephrine or serotonin) throughout the experiment were used for the evaluation of the relaxation to acetylcholine.

From each artery, four segments were used. The contraction to phenylephrine (PE; 10 nM to 100 µM; mesenteric arteries) or serotonin (SE; 10 nM to 100 µM; coronary arteries) was obtained. Subsequently, these segments were precontracted with PE or SE (both 10 µM) to a level of about 70% of E_max_ [31], [32] and endothelium-dependent relaxation to acetylcholine (ACh; 10 nM to 300 µM) was assessed. Precontraction levels were found stable throughout the protocol (data not shown). The maximal endothelium-independent relaxation was assessed by addition of sodium nitroprusside (SNP; 0.1 mM).

### Drugs

Phenylephrine, serotonin, acetylcholine and sodium nitroprusside were purchased from Sigma (Sigma Aldrich, The Netherlands) and were dissolved in deionized water. Dimethyl sulfoxide (DMSO) was purchased also from Sigma (Sigma Aldrich, The Netherlands). FTY720 and SEW2871 were purchased from Tocris Bioscience (United Kingdom) and were dissolved in 125 µl DMSO and diluted with 375 µl saline (0.9% NaCl).

### Statistical analysis

Data are expressed as mean ± SEM. Contractile responses are presented in mN and relaxations to ACh and SNP were calculated as a percentage of preconstriction. Concentration-response curves were plotted with SigmaPlot for Windows version 10.0 (Systat Software, Chicago, Illinois, U.S.A.). Concentration-response curves to PE, SE and ACh were characterized by area under the curve (AUC) in arbitrary units (au). AUC values were calculated by SigmaPlot. All statistical analyses were done with SPSS 16.0.2 for Windows (SPSS Inc. Headquarters, Chicago, Illinois, U.S.A.). Levene's test was used to confirm the homogeneity of variance. If data were normally distributed, differences were evaluated using Student's t-test (independent sample t-test) or One-Way ANOVA with posthoc Bonferroni test, where applicable. Differences between concentration-response curves were evaluated by Repeated-Measures ANOVA followed by Bonferroni test. If normality test failed, results were analyzed using Mann-Whitney Rank Sum test. Differences were considered significant at *P*<0.05.

## Results

### Blood gas analysis and hemodynamics

The data on blood gas analysis are given in Table 1. In Sham and CPB groups at baseline all parameters were in the normal range. During ECC in CPB, the pH, bicarbonate (HCO^−^_3_) level, and base excess (BE) was decreased, while pCO_2_ was increased. These findings may represent a moderate respiratory acidosis (Table 1). The hematocrit was reduced in CPB, likely due to hemodilution with the priming solution. After ECC, all parameters, except hematocrit, restored to baseline values and were not different between Sham and CPB (Table 1). The parameters of blood gas analysis in the groups pre-treated with FTY720 and SEW2871 were similar to the corresponding Sham and CPB groups (Table 1).

**Table 1:** Data on blood gas analysis and hematocrit in Sham and CPB groups.

Sham	Vehicle Before ECC	Vehicle 15 min ECC	Vehicle 45 min ECC	Vehicle After ECC	FTY720 Before ECC	FTY720 15 min ECC	FTY720 45 min ECC	FTY720 After ECC	SEW2871 Before ECC	SEW2871 15 min ECC	SEW2871 45 min ECC	SEW2871 After ECC
pH	7.43±0.05	7.45±0.05	7.46±0.07	7.41±0.06	7.4±0.02	7.4±0.03	7.4±0.03	7.36±0.03	7.4±0.02	7.4±0.02	7.37±0.03	7.35±0.01
pCO_2_	4.74±1.0	4.51±0.8	4.68±1.0	5.15±0.9	4.8±0.3	4.6±0.3	5.0±0.3	5.6±0.4	4.7±0.3	4.7±0.3	5.7±0.5	5.9±0.3
HCO_3_	17.4±4.7	16.6±3.9	17.7±4.2	16.9±4.6	22.1±0.3	22.3±0.5	23.8±0.5	23.8±0.5	21.5±0.8	21.5±0.7	23.9±0.7	23.9±0.5
BE	22.9±1.5	23.3±1.6	24.7±1.6	24.4±2.2	−1.8±0.4	−0.84±0.5	0.05±0.3	−1.4±0.4	−2.1±0.6	−1.9±0.5	−0.7±0.7	−1.2±0.4
Hctc	0.41 ±0.02	0.41±0.03	0.42±0.03	0.43±0.03	0.41±0.02	0.41±0.01	0.36±0.03	0.32±0.02	0.36±0.01	0.41±0.02	0.32±0.01	0.32±0.01

CPB	Vehicle Before ECC	Vehicle 15 min ECC	Vehicle 45 min ECC	Vehicle After ECC	FTY720 Before ECC	FTY720 15 min ECC	FTY720 45 min ECC	FTY720 After ECC	SEW2871 Before ECC	SEW2871 15 min ECC	SEW2871 45 min ECC	SEW2871 After ECC
pH	7.42±0.05	7.38±0.07**a**	7.32±0.08**a**	7.37±0.05	7.37±0.03	7.3±0.01**b**	7.3±0.02**b**	7.33±0.01	7.35±0.01	7.3±0.03	7.3±0.01	7.4±0.03
pCO_2_	4.77±0.8	5.07±1.1**a**	5.72±1.0**a**	5.22±0.8	5.0±0.4	6.2±0.2**b**	6.9±0.4**b**	5.8±0.2	5.4±0.2	5.7±0.3	6.6±0.2	5.6±0.5
HCO_3_	19.6±7.0	34.1±17.1**a**	35.8±18.3**a**	19.4±10.2	21.3±0.3	21.9±0.2	22.3±0.7	22.3±0.6	22.5±0.4	21.7±0.4	23.7±1.0	23.7±0.7
BE	22.6±2.3	21.5±3.6	21.6±2.4**a**	22.4±1.7**a**	−3.2±0.3**b**	−3.6±0.2**b**	−4.2±0.7**b**	−3.0±0.6**b**	−2.3±0.4	−3.2±0.7	−2.3±0.9	1.0±0.9
Hctc	0.41±0.02	0.31±0.02**a**	0.31±0.03**a**	0.36±003	0.36±0.01	0.21±0.06**b**	0.22±0.02**b**	0.33±0.03	0.34±0.07	0.22±0.02**c**	0.28±0.02**c**	0.38±0.02**c**

Abbreviation: CPB, cardiopulmonary bypass; ECC, extracorporeal circulation; BE, Hctc; Before ECC, 15 min before the start of extracorporeal circulation; 15 min ECC, 15 min after the start of extracorporeal circulation; 45 min ECC, 15 min before the end of extracorporeal circulation; after ECC, 15 min after the end of extracorporeal circulation.

**a**-*P<0.05* vs Sham untreated, t-test; **b**-*P<0.05* vs Sham FTY720, t-test; **c**-*P<0.05* vs Sham SEW2871, t-test.

**Figure 1:**
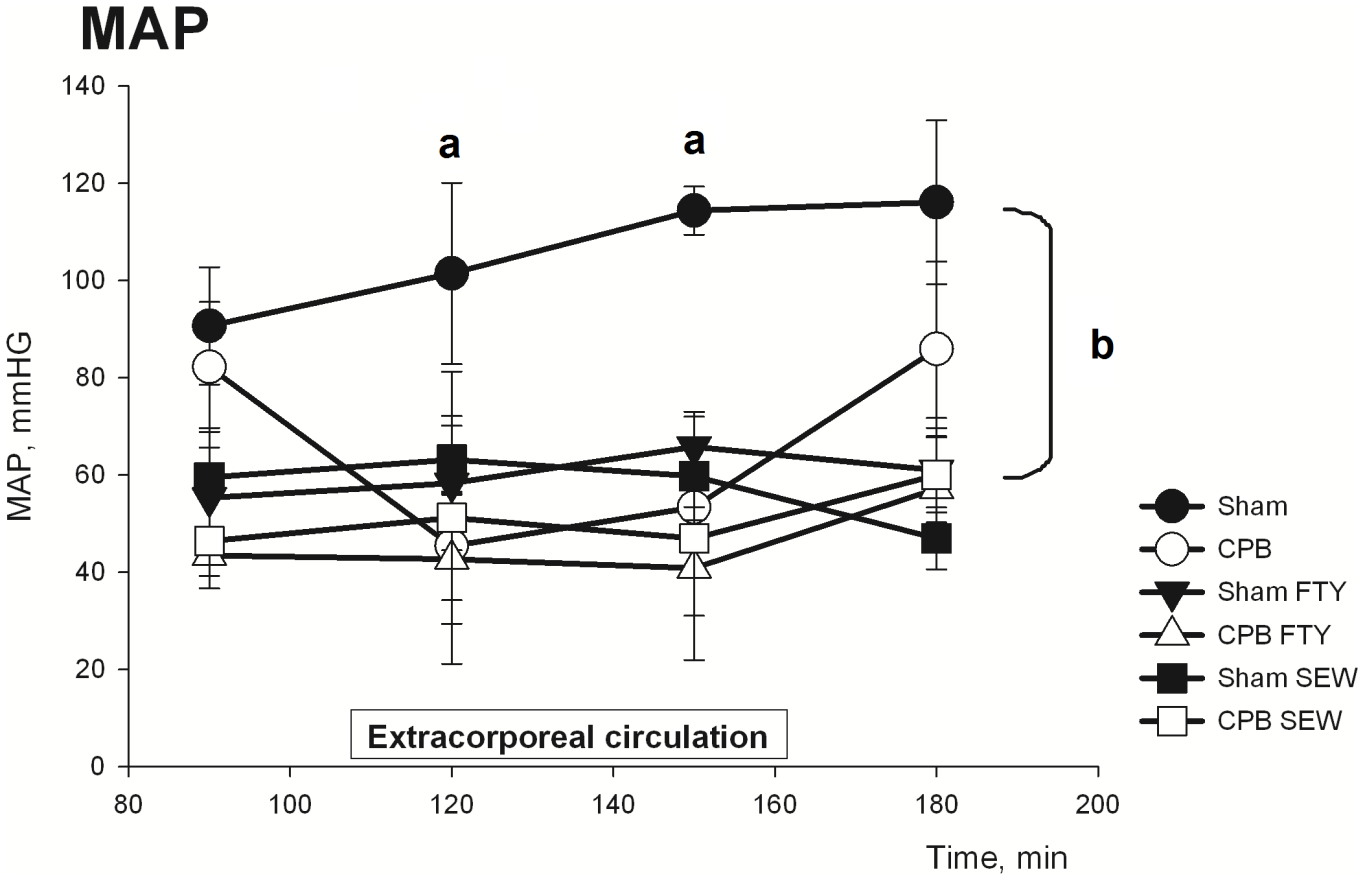
Data on mean arterial pressure in Sham and CPB groups during the experimental protocol. Abbreviations: MAP, mean arterial pressure; CPB, cardiopulmonary bypass. Data are mean±SEM. **a**- indicates P<0.05, t-test; **b**- indicates P<0.05, Repeated Measurements Anova, Bonferroni test.

At baseline, the MAP was not different between Sham and CPB. During ECC in CPB animals, the MAP dropped and remained significantly decreased until re-institution of normal circulation (*P*<0.05, Figure 1). In Sham animals, both FTY720 and SEW2871 caused a pronounced and significant decrease of MAP as compared with vehicle-treated Sham and untreated Sham (*P*<0.05, Figure 1). However, while CPB substantially lowered MAP, this was not further lowered by FTY720 and SEW2871. Moreover, after weaning from CPB MAP recovered in Sham CPB, but remained substantially decreased in Sham and CPB animals treated with FTY720 and SEW2871 (Figure 1).

### Blood cell counts

Table 2 shows the blood cell counts before, during and after the experimental procedure. All values are corrected for the hematocrit in order to discard the effects of hemodilution. Both Sham and CPB procedures evoked a substantial neutrophilia at the end of extracorporeal circulation period, which returned towards baseline levels after 24 hours of recovery (Table 2). Moreover, the increase in Sham was significantly lower compared with CPB (*P*<0.05, Sham-Vehicle vs CPB-Vehicle, Repeated measurements ANOVA followed by LSD test). A similar pattern of post-procedure neutrophilia was observed in Sham and CPB animals treated with FTY720 or SEW2817 (Table 2).

**Table 2:** Neutrophils, Lymphocyte and Monocyte counts in blood.

Vehicle	Sham baseline	Sham start ECC	Sham end ECC	Sham 24 h	CPB baseline	CPB start ECC	CPB end ECC	CPB 24 h
Neutrophils	1.9±0.3	1.7±0.4	6.5±1.2**a**	3.5±0.9	2.4±0.5	6.5±1.2	10.3±2.4**a**	6.6±0.5
Lymphocytes	23.5±3.2	14.7±1.2	18.7±2.4	16.7±1.2	24.3±1.5	37.6±2.3	34.9±2.2	9.8±2.1**b**
Monocytes	0.7±0.3	0.31±0.1	0.5±0.3	0.8±0.2	1.2±0.3	2.3±1.1	1.8±0.5	2.3±1.2

FTY-720	Sham baseline	Sham start ECC	Sham end ECC	Sham 24 h	CPB baseline	CPB start ECC	CPB end ECC	CPB 24 h
Neutrophils	1.2±0.04	3.5±1.0	6.7±0.5**c**	4.9±0.5	2.3±0.5	7.8±1.1	3.0±0.3	6.0±0.5
Lymphocytes	24.4±2.8	4.8±0.5**d**	4.8±0.6**d**	1.4±0.5**d**	21.2±1.5	8.3±1.1**e**	6.2±1.6**e**	2.8±0.9**e**
Monocytes	1.0±0.1	0.5±0.2	1.4±0.4	0.9±0.5	0.7±0.4	0.7±0.4	1.0±0.6	0.7±0.3

SEW2871	Sham baseline	Sham start ECC	Sham end ECC	Sham 24 h	CPB baseline	CPB start ECC	CPB end ECC	CPB 24 h
Neutrophils	2.5±0.6	2.3±0.6	11.9±1.5**f**	6.5 ±0.9	3.3±0.8	4.8±1.0	5.7±0.8	6.3±1.2
Lymphocytes	20.9±1.2	18.2±3.0	19.2±9.6	16.5±4.5	27.6±1.9	40.6±7.2	32.0±3.8	11.4±3.4**g**
Monocytes	1.0±0.1	0.8±0.2	0.35± 0.1	0.7±0.3	0.5±0.2	0.4±0.1	1.4±0.4	0.8±0.3

Values were normalized to Hctc (10^9^/l at a Hctc of 0.4). Baseline values were obtained prior to administration of compounds. Abbreviations: CPB, cardiopulmonary bypass; baseline, 15 min after injection of the experimental compound of the vehicle; start ECC, 15 min before extracorporeal circulation; end ECC, 15 min after extracorporeal circulation; 24 h, scarification at 24 h later the end of the extracorporeal circulation.

a- p<0.05 vs Baseline, t-test; b- p<0.05 untreated CPB 24 h vs all other CPB untreated groups, One-Way ANOVA, Bonferroni test; c-p<0.05 vs Sham FTY720 baseline, One-Way ANOVA on ranks, Dunn's Method (SigmaStat); d- p<0.05 vs Sham FTY720 baseline, One-Way ANOVA, Bonferroni test; e- p<0.05 vs CPB FTY720 baseline, One-Way ANOVA, Bonferroni test; f- p<0.05, vs Sham SEW2871 baseline, One-Way ANOVA, Bonferroni test;

g- p<0.05 CPB SEW2871 24 h vs all other CPB2871 groups, One-Way ANOVA, Bonferroni test.

In Sham animals, lymphocyte count seemed to decrease gradually during the procedure and 24 h recovery. In CPB, lymphocyte count increased during the procedure, which was followed by a decrease of more than 50% after 24 h of the recovery (*P*<0.05, One Way ANOVA followed by Bonferroni test). As expected, treatment with FTY720 induced a pronounced lymphopenia both in Sham and CPB (*P*<0.05, Table 2). This effect was present already at the start of the extracorporeal circulation and maintained throughout the 24 hour recovery period, at which point the number of circulating lymphocytes was decreased to 5–10% of the preoperative value (Table 2). Treatment with SEW2871 induced similar dynamics in circulating lymphocytes as observed in untreated CPB and SHAM animals (*P*>0.05, Table 2). Finally, monocyte count did not differ significantly between groups and was unaffected by CPB.

Thus, both Sham and CPB increased the number of circulating neutrophils. CPB increases circulating lymphocyte number during the procedure, which was followed by a substantial decrease at 24 hour of recovery. At 24 h recovery, FTY720 further decreased the number lymphocytes by 90–95% as compared with the preoperative value. In contrast, SEW-2871 did not further decrease the circulating lymphocyte (additional to lymphopenia registered in untreated CPB group).

### IL-6 plasma concentration

The plasma IL-6 concentration in the untreated and FTY720-treated groups are summarized in Table 3. In the Control group the plasma level of IL-6 was not detected. IL-6 concentration was slightly increased in Sham from 1 h up to 1 day of the recovery, whereas CPB caused a significantly increase in IL-6 plasma concentration at 1 h of recovery compared with Sham (P<0.05, t-test), which returned to Sham level at 1 day of recovery. Notably, the pre-operative treatment with a single application of the FTY-720 did not affect the plasma concentration profile of IL-6 in both Sham and CPB animals.

**Table 3:** The IL-6 plasma concentration (mmol/L) in untreated controls, Sham and CPB groups Vehicle and treated with FTY-720 after 1 hour and 24 hours recovery period.

Group	1 hour recovery	24 h recovery
**Healthy controls**	Not detected	–
**Sham-Vehicle**	2.8 ± 2.2	5.7 ± 3.3
**CPB-Vehicle**	4449 ± 589	2.7 ± 1.8
**Sham FTY-720**	4.6 ± 2.2	5.6 ± 5.6
**CPB FTY-720**	3793 ± 593**a**	9.1 ± 5.2

Abbreviations: CPB, cardiopulmonary bypass; 24 h, scarification at 24 h later the end of the extracorporeal circulation.

**a**- p<0.05 vs CPB, t-test.

### Effects of CPB and treatment with FTY720 and SEW2871 on contractile vascular reactivity

#### Mesenteric artery

**Figure 2:**
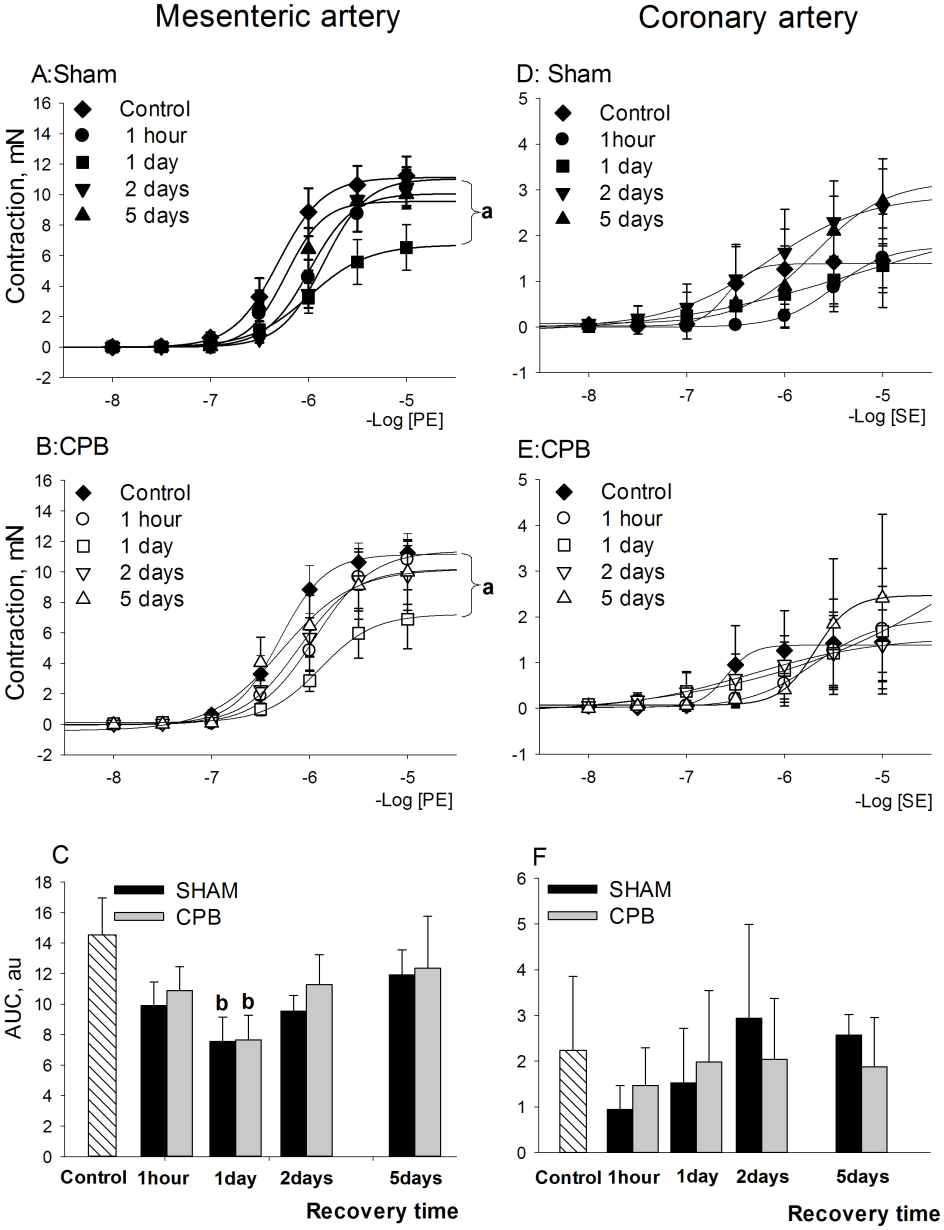
Contractile reactivity after short and long-term recovery following Sham or CPB. *Left panels*: contractile reactivity of mesenteric arteries to PE. **A**: concentration-response curves for Sham groups, mN; **B**: concentration-response curves for CPB groups, mN; **C**: AUC values of the contractile responses to PE, au. *Right panels*: contractile reactivity of coronary arteries to SE. **D**: concentration-response curves for Sham groups, mN; **E**: concentration-response curves for CPB groups, mN; **F**: AUC values of the contractile responses to SE, au. Abbreviations: CBP, cardiopulmonary bypass; PE, phenylephrine; SE, serotonin. Data are mean±SEM. **a**- indicates P<0.05 vs Control-Vehicle, Repeated measurements ANOVA, Bonferroni test; **b**- indicates P<0.05 vs CPB-Vehicle, One Way ANOVA, Bonferroni test.

Full concentration-response curves to phenylephrine (PE) were constructed in mesenteric artery (Figure 2, left panels) and data on maximal responses (Emax) are specified in Table 4. Both Sham and CPB procedures reduced the contractility of mesenteric artery most prominently at 1 day of recovery, whereas it returned to the baseline level at the 5^th^ post-procedure day (Figure 2A,2B; *P*<0.05 vs Control, Repeated Measurements Anova, Bonferroni test). The decrease in contractility at 1 day recovery is also reflected by a decrease in the area under the curve (Figure 2C; *P*<0.05 vs Control, One-Way Anova, Bonferroni test) and Emax (Table 4; *P*<0.05, One-Way Anova, Bonferroni test).

**Table 4:** Responses to phenylephrine (in mesenteric arteries), serotonin (in coronary artery), acetylcholine and sodium nitroprusside in Control, Sham and CPB following different recovery periods.

Mesenteric artery	PE, Emax, mN	PE, Emax, mN	PE, Emax, mN	ACh, Emax,%	ACh, Emax,%	ACh, Emax,%	SN, Emax,%	SN, Emax,%	SN, Emax,%
	Control	Sham	CPB	Control	Sham	CPB	Control	Sham	CPB
**Recovery**	11.3±1.3			58.6±6.6			93.9±3.0		
**1 hour**		9.5±1.3	10.8±0.9		70.8±4.7 **b**	82.0±4.6**b**		95.2±6.0	97.7±0.4
**1 day**		6.5±1.5**a**	7.9±1.9**a**		60.5±6.1	66.8±14.4		93.8±1.8	98.5±1.5
**2 days**		10.4±1.2	9.8±2.3		58.8±11.0	64.5±9.6		94.6±3.4	91.4±4.2
**5 days**		10.0±1.0	10.0±2.1		41.0±11.1	40.54±12.9		92.4±4.0	97.5±2.27

Coronary artery	SE, Emax,mN	SE, Emax,mN	SE, Emax,mN	ACh, Emax,%	ACh, Emax,%	ACh, Emax,%	SN, Emax,%	SN, Emax,%	SN, Emax,%
	Control	Sham	CPB	Control	Sham	CPB	Control	Sham	CPB
**Recovery**	1.4±0.5			54.8±18.6			116.8±7.8		
**1 hour**		1.5±0.2	1.7±0.4		58.0±3.1	72.6±4.2**b**		99.1±7.0	100.7±6.4
**1 day**		1.2±0.2	1.9±0.5		47.6±8.0	59.9±8.4		110.5±4.6	110.4±6.7
**2 days**		3.4±0.7	1.4±0.3		53.8±8.0	41.9±7.5		115.7±5.5	139.5±19.2
**5 days**		2.8±0.4	2.4±0.7		39.9±9.3**b**	49.8±9.3		107.3±10.6	112.0±6.4

Data are given as mean±SEM. Abbreviations: Ach, acetylcholine; CPB, cardiopulmonary bypass; Emax, the maximal response; PE, phenylephrine; SE, serotonine; SN, sodium nitroprusside; %- percent of the relaxation.

**a**-P<0.05 vs Control, One-Way ANOVA with post-hoc Bonferroni test; **b**- p<0.05 vs Control, t\test.

**Figure 3:**
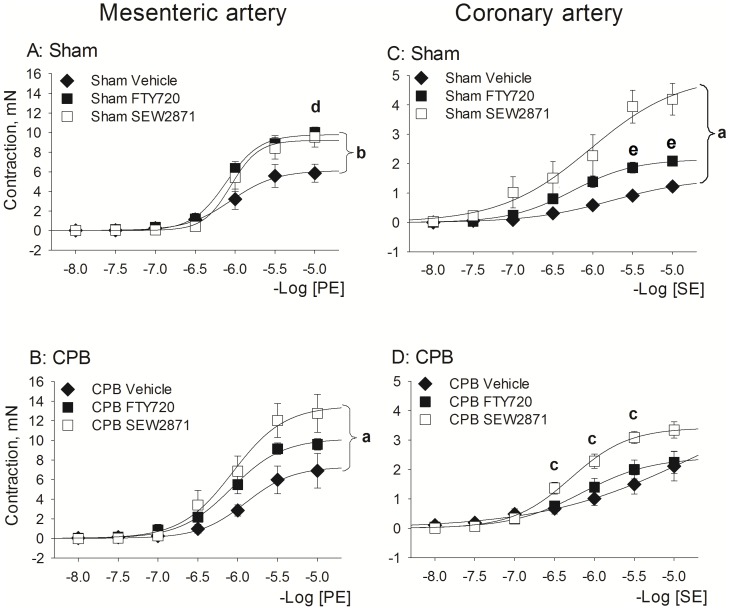
Effect of S1P receptor agonist pretreatment with FTY720 and SEW2871 on contractile responses at 1 day following recovery from Sham or CPB. *Left Panels*: responses to PE in mesenteric arteries. *Right panels*: responses to SE in coronary arteries. Abbreviations: CPB, cardiopulmonary bypass; PE, phenylephrine; SE, serotonin. Data are mean±SEM. **a**- indicates P<0.05 SEW2871 vs Vehicle, Repeated Measurements ANOVA, Bonferroni test (P = 0.007 for coronary artery and P = 0.037 for mesenteric artery); **b**- indicates P = 0.018 FTY720 vs Vehicle, Repeated Measurements ANOVA, Bonferroni test; **c**- indicates P = 0.04, SEW2871 vs Vehicle, t-test; **d**- indicates P = 0.028 SEW2871 vs Vehicle, t-test; **e**- indicates P = 0.01 FTY720 vs Vehicle, t-test.

The effect of treatment with FTY720 and SEW2871 on mesenteric contraction is presented in Figure 3 (left panels). Treatment with FTY720 and SEW2871 increased the contractile response to PE to a similar extent in Sham animals (FTY720: *P* = 0.018 vs Vehicle group; SEW2871: *P* = 0.06 vs Vehicle group, Repeated Measurements ANOVA, Bonferroni test). Moreover, both treatments also increased PE-induced contraction in CPB, although the effect of SEW2871 (*P* = 0.037 vs Vehicle group, Repeated Measurements ANOVA, Bonferroni test) seemed more prominent than that of FTY720 (*P* = 0.7 vs Vehicle group, Repeated Measurements ANOVA, Bonferroni test).

Thus, both CPB and Sham procedures inhibit vascular contractile responsiveness most prominently at 1 day of recovery, suggesting that this is due to anesthesia/cannulation rather than extracorporeal circulation. Both FTY720 and SEW2871 seem effective in improving the contractile vascular function at 1 day of recovery.

#### Coronary artery

Full concentration-response curves to serotonin (SE) were constructed in coronary artery (Figure 2, right panels) and data on maximal responses (Emax) to SE are given in Table 3. Both Sham and CPB caused a right-shift in SE concentration–response curves of coronary artery after short-term recovery (i.e. at 1 h and 1 day recovery). The effect of treatment with FTY720 and SEW2871 is presented in Figure 3 (right panels). SEW2871 significantly increased the contractile response to SE in both Sham and CPB as compared with corresponding Vehicle-treated group (Sham: *P*<0.05, Repeated Measurements ANOVA, Bonferroni test; CPB: *P*<0.05, t-test). FTY720 showed a tendency to normalize contractile responsiveness in Sham (*P*<0.05, t-test), but was without effect in CPB.

Thus, Sham and CPB inhibit contractile vascular reactivity of coronary artery to SE at 1 day of recovery. Treatment with SEW2871 normalized this loss of contractility, whereas FTY720 shows only moderate effectiveness.

### Effects of CPB and treatment with FTY720 and SEW2871 on vascular relaxation

#### Mesenteric artery

**Figure 4:**
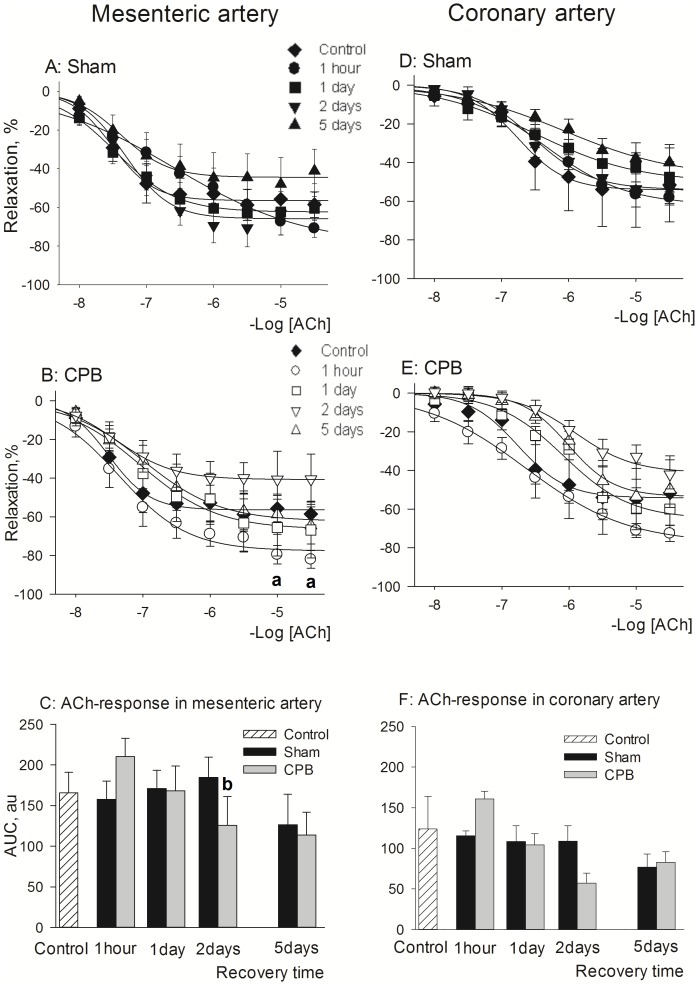
Relaxant reactivity after short and long-term recovery following Sham or CPB. *Left panels*: relaxant reactivity of mesenteric arteries to ACh. **A**: concentration-response curves for Sham groups, % of relaxation; **B**: concentration-response curves for CPB groups, % of relaxation; **C**: AUC values of the relaxant responses to ACh, au. *Right panels*: relaxant reactivity of coronary arteries to ACh. **D**: concentration-response curves for Sham groups, % of relaxation; **E**: concentration-response curves for CPB groups, % of relaxation; **F**: AUC values of the relaxant responses to ACh, au. Abbreviations: CBP, cardiopulmonary bypass; PE, phenylephrine; SE, serotonin. Data are mean±SEM. **a**- indicates P<0.05 vs Control-Vehicle, t-test. **b**- indicates P<0.05 vs Sham 2 days, t-test.

Full concentration-response curves to acetylcholine (ACh) were constructed in mesenteric artery (Figure 4, left panels) and data on maximal response (Emax) to acetylcholine and sodium nitroprusside are given in Table 4. The Sham procedure did not affect the vascular relaxation to ACh (Figure 4A, Table 4). In CPB, maximal ACh-mediated relaxation was enhanced at 1 h of recovery, however with a later reduction at 2 days (Figure 4B,C). Generally, it was found that in mesenteric artery relaxant reactivity to ACh was similar in Control and Sham/CPB rats, suggesting that its endothelial function at 1 day of recovery was intact (Figure 4). Endothelium-independent relaxation was unaffected in both Sham and CPB since the relaxation to sodium nitroprusside was similar in all groups (Table 4).

**Figure 5:**
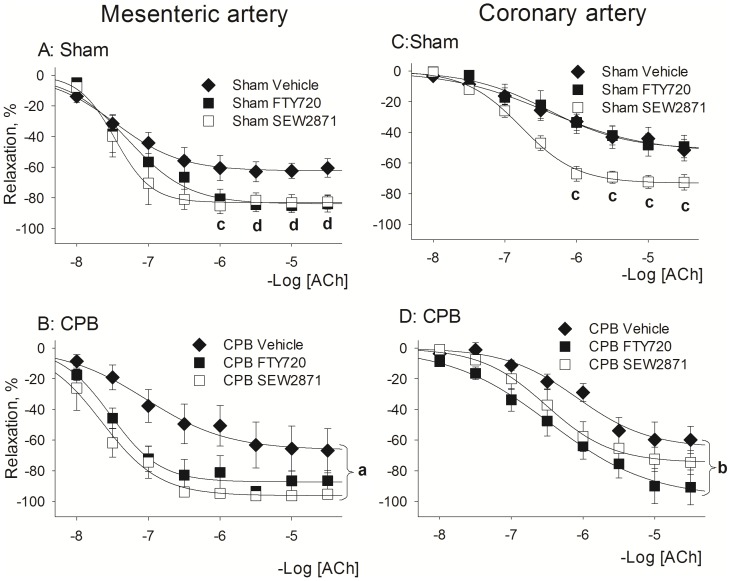
Effect of S1P receptor agonist pretreatment with FTY720 and SEW2871 on relaxant reactivity at 1 day following recovery from Sham or CPB. ACh-induced relaxation was studied in mesenteric arteries (left panels A and B) and coronary arteries (right panels C and D). Abbreviations: CPB, cardiopulmonary bypass, ACh, acetylcholine. Data are mean±SEM. a- indicates P = 0.026 SEW2871 vs Vehicle, Repeated Measurements ANOVA, Bonferroni test; b- indicates P = 0.018 FTY720 vs Vehicle Repeated Measurements ANOVA, Bonferroni test; c- indicates P = 0.03 SEW2871 vs Vehicle, t-test; d- indicates P = 0.03 FTY720 vs Vehicle, t-test.

Effects of the treatment with FTY720 and SEW2871 on vascular relaxant reactivity are given in Figure 5. Interestingly, both treatments seem to enhance total relaxation to ACh by about 25%, both in Sham and CPB. However, only in in CPB rats treated with SEW2871 this effect reached statistical significance (Figure 5; *P* = 0.026; CPB-SEW2871 vs CPB-Vehicle, Repeated Measurements ANOVA, Bonferroni test).

#### Coronary artery

Full concentration-response curves to acetylcholine (ACh) were constructed in coronary artery (Figure 4, right panels) and data on maximal response (Emax) to acetylcholine and sodium nitroprusside are given in Table 4. During the 5 days recovery period, the total ACh mediated dilatation was mainly unaltered in both Sham and CPB compared to control rats (Figure 4D–F), while in CPB the maximal response to ACh was significantly increased after 1 h of recovery (Table 4). Endothelium-independent relaxation was unaffected since the relaxant responses to sodium nitroprusside were similar in all groups (Table 4).

Effects of the treatment with FTY720 and SEW2871 on vascular relaxant reactivity are given in Figure 5 (right panels). Both treatments displayed a tendency to enhance the total relaxation to Ach by about 20%, while their efficacy was different in Sham and CPB. SEW2871 was more effective in Sham (P<0.05, vs Sham-Vehicle, t-test; Emax in Sham SEW2871: 72.4±4.2%; Emax in Sham Vehicle: 51.5±7.0%) and FTY720 was significantly more effective in CPB (*P* < 0.05, vs CPB-Vehicle, Repeated Measurements ANOVA, Bonferroni test).

Thus, in both mesenteric and coronary artery during 5 days of recovery, the relaxant reactivity was similar in Control and Sham/CPB rats. Nevertheless, both FTY720 and SEW2871 seemed to improve the vascular relaxant response to ACh both in Sham and CPB.

## Discussion

The present work studied the effects of treatment with FTY720 and SEW2871, two different S1P receptor agonists, on vascular reactivity in an experimental rat model of cardiopulmonary bypass. In accordance with the proposed mechanisms of action, we found FTY720, but not SEW2871, lowers circulating lymphocyte count. Both FTY720 and SEW2871 were found to cause a pronounced reduction in blood pressure during the experimental protocol in both Sham and CPB. Further, we found the CPB-related surgical procedures and/or extended anesthesia to induce extensive and protracted changes in the contractility of small vessels, as contractility of both mesenteric and coronary artery was inhibited, which was most prominent at 1 day of recovery. Preoperative administration of both FTY720 and SEW2871 normalized the vascular reactivity by increasing the vascular responsiveness to PE (mesenteric artery) and SE (coronary artery). In contrast, the vascular relaxation to ACh was essentially unchanged in Sham and CPB during 5 days of recovery. However, both FTY720 and SEW2871 increased ACh induced vascular relaxation in both vascular beds by about 20–25%. Thus, both compounds evoked hypotension, improved vascular contractile and relaxant responsiveness, yet differentially decreased the amount of lymphocytes in the peripheral blood. These data suggest that the observed systemic vascular treatment effects of FTY720 and SEW2871 were independent from lymphopenia but rather involved the modulation of vascular S1P1 receptors.

### Vascular reactivity after CPB

Vascular dysfunction contributes to multiple organ dysfunction syndrome after CPB and is therefore considered to be a relevant target of therapy. We found that contractility of both mesenteric and coronary arteries was impaired by both experimental procedures (i.e. Sham and CPB), most prominently at one day of recovery. The finding that both Sham and CPB negatively affects the vascular contractile function, suggests that the minor surgical procedures and/or extended anesthesia induce these extensive and protracted changes in the contractility of small vessels, rather than ECC. In addition, we found that the relaxant vascular function was much less affected: only the relaxant function of mesenteric arteries was briefly affected following CPB and only minor effects of surgery (i.e. sham and CPB) were found on the relaxant function of the coronary arteries. Collectively, our data suggest that anesthesia and cannulation have a long-lasting impact on the vasoresponsiveness of small arteries, which warrants further investigation into its mechanism and the contribution of different types of anesthetics.

### Influence of S1P-receptor agonists on the amount of lymphocytes

Although S1P is of importance in the entire human body, it is a major regulator of the vascular and immune system. In this respect, the immunomodulatory effects of S1P agonists have been associated with the inhibition after S1P receptor activation of the egress of lymphocytes from secondary lymphoid organs to peripheral blood [33]. FTY720 is considered a potent non-selective S1P receptor agonist [9]–[12]. Upon binding to the S1P receptor, FTY720 induces internalization of the receptors and induces their degradation, leading to a downregulation in the number of S1P receptors [34]. By reducing the number of receptors, FTY720 can be considered as a functional antagonist. SEW2871 is a highly selective S1P_1_ receptor agonist that in contrast to FTY720 does not induce degradation of its receptor after internalization and is thereby not able to downregulate S1P_1_ receptors [34]–[35]. We found that FTY720 induced a profound decrease in the amount of circulating lymphocytes, but not in neutrophils or monocytes. FTY720-induced lymphopenia was present at the onset of extracorporeal circulation (approximately 4 hour after the injection) and lasted for (at least) 24 hours thereafter. In contrast, the number of circulating lymphocytes was not affected by SEW2871. Further, CPB induced a marked systemic inflammatory response as evidenced with the increase in plasma IL-6, which was unaffected by the pre-operative treatment with the single dose of FTY-720. Collectively, the above data strongly indicate that the reported vascular effects of FTY720 and SEW2871 are independent of its effect on immune cells.

### Effect on Mean Arterial Blood Pressure

Both FTY720 and SEW2871 decreased the MAP significantly during the operative period, which most likely involves their action on S1P_1_ receptors. Indeed, activation of S1P_1_ (and S1P_3_) receptors on endothelium enhances the production of nitric oxide and has vasodilatory effects [36]. Alternatively, the reduction in MAP by FTY720 might involve signaling through S1P_3_ receptors, as described previously [37] or it might be result from S1P_3_ receptors induced bradycardia [38]. We did not record heart rate and therefore we cannot report whether the pretreatment with the experimental compound also caused bradycardia in our experimental settings. Further, FTY720 may inhibit the secretion of prostanoids, including contractile prostaglandins such as thromboxane A_2_, via a receptor-independent inhibition of phospolipase A_2_, a key enzyme of arachidonic acid-derived eicosanoid formation [39]. Taken together, both FTY720 and SEW2871 similarly decreased the blood pressure during surgery, which suggests it is mediated mainly by S1P_1_ receptor mediated signaling.

### Effect of S1P receptor agonists on contractile vascular reactivity

The depressed mesenteric contractility was used to evaluate the potential of agonists of S1P receptors to modulate vascular dysfunction in CPB. We show that injection of a S1P_1_ receptor agonist improves the vascular contractile and relaxant function of mesenteric and coronary arteries and thereby, at least in part, preclude the negative effects of surgery and CPB on these vessels. Despite the differential effects of FTY720 and SEW2871 on circulating lymphocytes in the present study, both compounds shared a similar effect on vascular reactivity and blood pressure, which suggests that the effects on the vascular function occur independently of immunological changes (i.e. lymphopenia), but rather involve modulation of vascular S1P receptors.

The comparable effect of both FTY720 and SEW2871 on vascular contractility suggests the involvement of S1P_1_ receptor-dependent mechanism, whereas vascular smooth muscle were reported to express mainly S1P2 and S1P3 receptors that mediate vasoconstriction [40]–[43] through activation of the phospholipase C, myosin light chain kinase and/or Rho-associated kinase-dependent inhibition of myosin light chain phosphatase [44]–[47]. However, other studies found the involvement of S1P1 receptors in the regulation of contractile reactivity [44]–[47] and S1P agonist were found recently to evoke vasoconstriction of the afferent renal arterioles via S1P1 and S1P2 receptors with involvement of the L-type voltage-dependent calcium channels [48]. Further, S1P induces COX-2 expression in vascular smooth muscle cells, which seems partially mediated via S1P1 receptors [49]. Thus, S1P1 receptor may be more prominently involved in VSMC regulation than reported so far. Alternatively, the similar effect of FTY720 and SEW2871 on VSM contractility may be dependent on S1P receptors expressed on the endothelium. Indeed, FTY720 evokes endothelium dependent VSMC contraction most likely via COX/thromboxane A2 route and sphingosine kinase [50]. Indeed, hyporeactivity of VSM to adrenomimetics may depend on changes in the endothelium, as found in septic shock [51]. Possibly, S1P1 agonist thus improve postoperative vascular contractile reactivity via modulation of this endothelium-dependent mechanisms.

### Effect of S1P receptor agonists on relaxant vascular reactivity

CPB was priory reported to cause a pronounced vascular dysfunction [52]–[54]. In several rat models, CPB induced endothelial dysfunction, mainly after 60–90 min of recovery [55], [56]. However, in our study the endothelium-dependent relaxation to ACh did not differ between Sham and CPB rats and was comparable to that in untreated Controls, demonstrating that endothelial function was intact at 1 day post-CPB. The lack of the endothelial vascular dysfunction in our study may be explained by differences between models and experimental protocols. The model we employed is a modification of the previously widely published model (Samarska et al. 2013, J Pharmacol Toxicol Methods). Our model differs on three points: miniaturization of the extracorporeal circuit to 15 ml avoids both blood transfusion and excessive hemodilution. Moreover, our model employs arterial inflow via the left common carotid artery to the aortic arch instead of inflow via the tail artery. All these factors may influence CPB-mediated endothelial dysfunction. In addition, differences with previously employed models regarding duration of the extracorporeal circulation and the postoperative recovery period may influence vascular reactivity. However, similar to the clinical setting, CPB, but not Sham, featured the activation of a systemic inflammatory response, demonstrating the fitness of our model to preclinically evaluate the potential of experimental therapeutic interventions.

Despite the lack of the endothelial dysfunction, FTY720 and SEW2871 both enhanced total relaxation to ACh in all groups, although augmentation of the endothelium-dependent relaxation was most pronounced in CPB. Most likely, in coronary artery, the enhanced relaxation to FTY720 and SEW2871 is caused by activation of S1P_1_ and/or S1P_3_ receptors on endothelial cells, which evoke the release of NO through the activation of eNOS [16]–[18]. On the other hand, relaxation of mesenteric arteries depends on EDHF rather than NO, which is only a minor mediator of its endothelium-dependent relaxation. Thus, in addition to amplification of the NO production, FTY720 and SEW2871 may have enhanced S1P_1_-receptor mediated increase in EDHF production or its action. Indeed, S1P was shown to activate large-conductance Ca(2+)-activated K(+) channel in a G-protein independent manner [47], but its effects on EDHF production needs further exploration. Nevertheless, because both experimental compounds exerted highly comparable effects, it seems conceivable that vascular effects are mainly mediated through S1P1 receptors.

In conclusion, we reveal that in the rat (1) CPB impairs vascular contractility in small arteries, which is due to both the surgical and anesthetic procedure and most prominent at one day following surgery, (2) CPB has a limited effect on vascular relaxant function and (3) vascular dysfunction following CPB is relieved by pre-operative treatment with FTY720 and SEW2871.
